# Healing Beams: Radiation and Radiotherapy in Novels, Poems, Music, Film, Painting

**DOI:** 10.1002/jmrs.70026

**Published:** 2025-10-21

**Authors:** Ad A. Kaptein, Jan W. Schoones, Yvette M. van der Linden, Brian M. Hughes

**Affiliations:** ^1^ Medical Psychology Leiden University Medical Centre Leiden the Netherlands; ^2^ Directorate of Research Policy Leiden University Medical Centre Leiden the Netherlands; ^3^ Department of Radiotherapy Leiden University Medical Centre Leiden the Netherlands; ^4^ School of Psychology University of Galway Galway Ireland

**Keywords:** art, health humanities, perceptions, radiation, radiation therapy

## Abstract

The utility of radiation and radiation therapy to treat patients with cancer is determined by more than just biomedical or physical factors: patient perceptions of radiation influence both uptake of and adherence to radiation therapy. Such perceptions have been represented in various genres of art and are identifiable in novels, poems, music, film and paintings. In this paper, we outline how radiation therapy has been represented in novels, poems, music, film and the visual arts. Adopting a narrative review approach, empirical research on patient perceptions of radiation therapy is briefly summarised, as are studies on behavioural interventions to help patients cope with radiation therapy. The potential applicability of music (music therapy), film (photovoice) and painting (art therapy) in patients having to undergo radiation therapy is briefly considered. Key findings pertain to improving psychological responses to radiation therapy (reduction in anxiety, depression, fatigue), medical outcomes (duration and perceived discomfort) and patient—healthcare provider communication. As health humanities is the overarching paradigm for research and clinical work in this domain, the examination of patient experiences of radiation therapy provides a novel and timely field of research and clinical work in radiation therapy and health psychology.

## Graphical abstract

Radiation and radiotherapy are perceived by patients and the general public as mysterious, dangerous and healing. The image of radiation and radiotherapy can be analysed by studying novels, poems, music, film and painting. Our paper reviews how radiation and radiotherapy are perceived by patients, the general public, as reflected in various art genres. Care in radiotherapy may benefit from incorporating these perceptions in medical management of patients required to undergo radiation and radiotherapy.

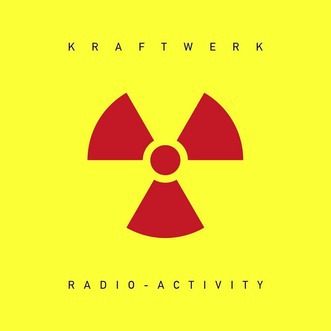

## 1 Introduction

Therapeutic medical management of patients with radiation therapy is perceived by the public at large as contributing positively to medicine and, by extension, to human health. At the same time, radiation is often seen as having a dangerous tone and connotations, and frequently elicits fear and confusion in humans, patients and wider society [1, 2, 3, 4]. Such anxiety might be addressable by invoking cultural means by which an understanding and appreciation of medical radiation can be cultivated. In this regard, health humanities, a rapidly developing area of research and clinical application, might be especially productive in combining biomedicine, psychosocial studies, humanities and the creative arts in order to explore a variety of human dimensions of health and illness. *Health humanities can be seen as* ‘a field concerned with understanding the human condition of health and illness in order to create knowledgeable and sensitive health care providers, patients, and family caregivers … drawing on the methodologies of the humanities, fine arts and social sciences to provide insight, understanding, and meaning to people facing illness including professional care providers, lay care providers, patients, policy‐makers and others concerned with the suffering of humans’ ([5], page 3).

Major journals in the health humanities publish papers on key aspects: exploring emotions (feelings) and cognitions (ideas, views) of patients regarding symptoms, medical care seeking and self‐managing (chronic) medical illness. Additional themes pertain to using various art genres in helping diagnose and manage medical conditions; studying the effects of therapeutic measures, which may include various art‐based interventions, to help patients cope with medical conditions. A major clinical area relates to helping to improve the quality of life (QOL) of patients and healthcare providers (HCP), with patient–HCP communication as a central concern. Additional support is presented in studies that have a diagnostic (e.g., [6, 7]) or therapeutic [8, 9, 10] focus.

Professional societies in the area of radiation therapy recommend the incorporation of patient perspectives on radiation therapy into medical management (e.g., ASTRO, American Society for Radiotherapy and Oncology; ESTRO, European Society for Radiotherapy and Oncology). Canadian radiation oncologist Charles Hayter expands on these issues from an insider's perspective in his book *Cancer Confidential: Backstage Dramas in the Radiation Clinic*, which provides a candid account of the politics and power plays that govern the position and status of radiation therapy treatments—and of radiation therapy departments—in hospital settings, as well as in society at large [11]. Similarly, Cooke and Awan outline the applicability of visual arts, theatre arts and literature and writing to the fields of radiation therapy and radiology with a view to improving the quality of life of patients [12].

Radiation, whether diagnostic or therapeutic, is usually represented in medical textbooks using diagrams, formulas and various types of medical imagery and technology. Successful medicine requires more, however, than mechanics and machinery. Patient perceptions of radiation and radiation therapy influence both uptake of and adherence to radiological diagnosis and radiation therapy treatment; as such, a deeper understanding of how human beings experience and process the prospect of radiation in medicine is needed to ensure that hesitation and anxiety are addressed. A recent paper in the *Journal of Medical Radiation Sciences* provides an excellent illustration of this view [13].

In the early 20th century, public perceptions of X‐ray machines, radium, radiation and their application to cancer largely evolved from hyped expectations (especially in the United States) about potential curative prowess to fearful beliefs that such methods posed a ‘subtle, cumulative, and insidious threat ([2], p. 587). Today, patients continue to report anxiety and fear when undergoing radiation therapy [13, 14]. Such anxieties and fears are frequently represented in mainstream cultural depictions of medical radiation, as seen in many novels, poems, musical pieces, films and paintings.

The representation of illness in art is therefore more than just a cultural curiosity. Artistic representations help to provide insights for theoretical models that themselves may be helpful for structuring interventions in behavioural medicine and health psychology [15]. Ideas such as illness narratives [16], explanatory models [17] and the so‐called common‐sense model of health behaviour [18] provide three examples of relevant theoretical approaches, with both research and clinical applicability. Bibliotherapy (i.e., a creative arts therapy that involves storytelling or the reading of specific texts [19])—along with music therapy, photovoice and art therapy—is a major clinical therapeutic approach that exemplifies health humanities in context [20]. Using an individual's relationship to the content of books and poetry and other written words as therapy, bibliotherapy partially overlaps with, and is often combined with, writing therapy (expressive writing). It is an active self‐help, brief, nonpharmacological intervention that draws on cognitive and behavioural therapy principles [21].

The aim of the current paper was to provide an overview of the empirical literature on the representation of radiation treatment in four art genres: literature (primarily novels and poems), music, film and paintings. We also consider the application of health humanities to therapeutic interventions in patients undergoing diagnostic and therapeutic procedures involving radiation therapy, especially as it impacts upon their well‐being, anxiety, self‐efficacy, fatigue and quality of life.

## 2 Methods

In this exploratory paper, material on theoretical and clinical work in the area of radiation therapy and health humanities was selected and assembled using various methods of data collation.

### References

2.1

Novels and poems were identified and examined using ‘LitMed’, the literature, arts and medicine database hosted by New York University (https://medhum.med.nyu.edu/browse). PubMed was also searched for articles on novels, poems, music, film and painting in relation to radiation therapy. A detailed search strategy was composed, and the query consisted of the combination of the following two concepts: * novels, poems, music, film, painting and * radiation therapy. For these two concepts, relevant keyword variations were used, not only keywords in the controlled vocabulary of PubMed (the MeSH database) but free text word variations of these concepts as well.

Three published volumes were also consulted. The first was *Medizin in der Literatur der Neuzeit* (‘Medicine in Modern Literature’, 2021) by von Engelhardt, which collates a phenomenal collection of novels on the theme of literature and medicine [19]. This volume was perused in order to identify novels in which radiation therapy plays a more or less prominent role. The second was *Krebs schreiben [‘Writing Cancer’]* (1997) by Moamai, which contains eight pages devoted to German novels in which radiation therapy is a major topic [3]. The third was *Cancer Poetry* (2015) by Twiddy [22], which presents a rich source of poems on cancer. In addition, journals on the theme of health humanities were studied (e.g., *Arts & Health*; *Hektoen International*; *Journal of Medical Humanities*; *Poetics Today*; *Psychology of Aesthetics*, *Creativity and Arts*), as were major medical journals that frequently carry health humanities content (e.g., *British Medical Journal*; *JAMA*; *JAMA Oncology*; *Lancet*; *New England Journal of Medicine*) and journals in the area of radiation therapy. The reference lists of identified papers were perused as a means of locating additional sources.

### 2.2 Music

The search strategy for music followed a similar approach. Literature searches were conducted on PubMed using search terms such as ‘music AND radiation, radiation therapy’, and relevant journals (such as *Psychology of Music*) and books (e.g., [23]) were consulted.

### 2.3 Films/Motion Pictures

Similarly, for film, literature searches were conducted via PubMed for ‘films AND radiation, radiation therapy’. Additional sources were identified by consulting the major review paper by de Fiore et al. [24] and the book *Cinema MD—a history of medicine on screen* (2020) by Wijdick [25].

### 2.4 Paintings

Literature searches in PubMed were conducted for ‘paintings AND radiation, radiation therapy’. The book *Medicine in Art* (2010) by Bordin and d'Ambrosio [26] and relevant journals were also consulted.

The searches described here are, by intention, exploratory and narrative rather than exhaustive. Our objective in this paper was to outline trends in the subject area and to set the scene for future research based on more systematic literature reviews [27]. The date range for the searches was 2016–2025.

## 3 Results

### References

3.1

Our searches identified several novels and works of poetry with prominently identifiable themes relating to radiation and radiation therapy.

#### 3.1.1 Novels

A selection of such novels is presented here. The first is *The Magic Mountain* by Thomas Mann (published in German in 1924 and in English in 1927), one of the most influential works of German literature [28]. In it, the protagonist Hans Castorp is allowed to see his own X‐ray of his thorax (quotes are given in Table 1).

**TABLE 1:** Quotations from selected novels.

The Magic Mountain by Thomas Mann [28] Hans Castorp was a little feverish with expectation, since until now no one had ever taken a look into his organic interior … (p. 207) … he saw that semidarkness, a kind of artificial twilight, reigned in the X‐ray room … the windows had been blacked out, daylight banned, and only a couple of electric bulbs were turned on … (p. 208); there was a peculiar odour here—a kind of stale ozone smell in the air … you could make out clinical apparatus of various sorts: glassware, switch boxes and tall vertical gauges, but also a camera‐like box on a rolling stand and rows of glass photographs plates set along the walls. You couldn't tell if you were in a photographer's studio, a darkroom, or an inventor's workshop and sorcerer's laboratory … (p. 211). … For 2 s the dreadful forces necessary to penetrate matter were let loose – a current of thousands of volts, one hundred thousand, Hans Castorp thought he had heard somewhere. Barely tamed for their purpose, these forces sought other outlets for their energy. Discharges exploded like gunshots. The gauges sizzled with blue light. Long sparks crackled along the wall. Somewhere a red light blinked, like a silent, threatening eye, and a vial behind the technician's back was filled with a green glow. … (p. 212): He dismounted, confused and dazed by what had happened to him, although he had not felt anything at all during the penetration … (p. 213); … And Hans Castorp saw exactly what he should have expected to see, but which no man was ever intended to see and which he himself had never presumed he would be able to see: **he saw his own grave** (emphasis added) … (p. 215); … and for the first time in his life he understood that he would die (p. 216). Get a life by Nadine Gordimer [29] Radiant. Literally radiant. But not giving off light as saints are shown with a halo. He radiates unseen danger to others from a destructive substance that has been directed to counter what was destroying him. Had him by the throat. Cancer of the thyroid gland. In hospital he was kept in isolation. Even that of silence: He had no voice for a while, mute. Vocal cords affected. He remains, he will be still, out of his control, exposing others and objects to what he emanates, whomever and whatever he touches’ … (pp. 3–4). … ‘the blinding dazzle of invading radioactive iodine’ … (p. 13), ‘dogs are put in quarantine quarters’ … (p. 16), ‘something he gave off, some kind of light you couldn't see’ … (p. 20), ‘taking care of the lit‐up leper‘… (p. 33), ‘the shaft of invisible light’ … (p. 43), ‘there are two Eras, BR, before discovery of the gland gone malignant, and AR, after radiance’ … (p. 51). Come d'Aria (Like air) by Ada d'Adamo [30] I felt the thickness of the wall between me and the one who, at a safe distance, controlled the switches to manoeuvre the mechanical arm according to plan. That wall represented the boundary between the ill and the healthy, between care providers and care receivers. At this side of the wall I felt boxed in, defenceless, mortally lonely (p. 24). In Gratitude by Jenny Diski [31] I was alone and had been made ready for the Elektra Linear Accelerator to perform its *danse macabre* around me. … Two massive arms were pulled out from the huge silver wheel set into the wall behind me, a third was already positioned above me. This one was circular with a mirrored surface in which I could see myself and the green line it projected on to me running vertically a couple of inches to the left of centre, marked by the indelible tattoo. … It wasn't designed to look what it did, or for its use to be understood with just one look (pp. 124–5). Cancer Ward by Alexander Solzhenitsyn [32] Through the square of the skin that had been left clear on his stomach, through the layers of flesh and organs whose names their owner himself did not know, through the mass of the toad‐like tumour, through the stomach and entrails, through the blood that flowed along his arteries and veins, through lymph and cells, through the spine and lesser bones and again through the hard wooden boards of the couch, through the 4‐centimetre‐thick floor‐boards, through the props, through the filling beneath the boards, down, down, until they disappeared into the very stone foundations of the building or into the earth, poured the harsh X‐rays, the trembling vectors of electric and magnetic fields, unimaginable to the human mind or else the more comprehensible quanta that like shells out of guns pounded and riddled everything in their path. (p. 77) Of course the X‐ray smashes everything it meets. Only normal tissues recover quickly, tumour tissues don't. (p. 78). They're battering me with X‐ray treatments, two sessions a day, 20 min each session at 300 rads, and although the pain I had is long forgotten, I have now come to know what nausea is. My friends, X‐ray nausea (or maybe it comes from the injections, everything here gets mixed)–you have no idea how loathsome it is. It gets you right in the chest and it gets on for hours. (p. 316).

The cover of *Get a Life* (2005) by Nadine Gordimer [29] states that its protagonist is a ‘committed environmentalist fighting a huge nuclear project with its danger of leaking radiation.’ In the novel itself, the protagonist's professional and personal lives become increasingly entangled when thyroid cancer raises its ugly head. The book consistently brings forward themes such as stigma, anxiety and control (quote in Table 1).


*Come d'Aria [Like air]* (2023) [30] tells the story of author Ada d'Adamo, who has fallen ill with breast cancer and must cope not only with her own disease but also the orphan disease (holoprosencephaly) experienced by her daughter. Radiation therapy is part of the medical management of her breast cancer (quote in Table 1).


*In Gratitude* (2016) by Jenny Diski presents the narrative of a woman who will imminently die because of lung cancer [31]. A prolific and quite famous English author, Diski writes about herself, her medical career and her healthcare providers with brutal honesty. Radiation therapy is part of the medical treatment she is subjected to (quote in Table 1).


*The Book About Blanche and Marie* (2004), while based on the lives and works of Marie Curie and one of her assistants, Blanche Whitman, is nonetheless ‘a novel’, as emphasised by its author Per Olov Enquist [33]. Radiation connects the two women across parallel stories: one about the discovery of radioactivity (focusing on Curie), the other about the work at the radiology department at the Salpêtrière hospital (concerning Whitman). In the novel, the latter woman gradually develops radiation injury, with hugely deforming consequences. (In a subsequent letter to the *Lancet*, a modern‐day neurologist criticised the historical correctness of these stories [34], perhaps unaware of the author's explicitly stated intention to produce a fictionalised account).

Physician/writer Richard Selzer covers a wide array of medical topics in his books. His short story *Luis* (1998) revolves around a 19‐year‐old boy, who searches the city dump of a large Brazilian city at night for anything valuable [35]. To his great delight, the boy stumbles upon a light‐emitting object, and he gleefully shares his find by giving small pieces of the object to family and friends. The object turns out to be a piece of discarded machinery from the radiation department of a large city hospital, a place where upper‐class patients receive radiation therapy for their cancers. As Selzer describes it, ‘inside the old machine a piece of metal gives off dangerous rays that kill human flesh’ (pp. 254–5). The boy Luis eventually dies; Selzer leaves the reader with a personalised story that situates radiation within a broader public health perspective.

Aleksandr Solzhenitsyn's autobiographical novel *Cancer Ward* (1972) [32] presents a moving account of the cancer journeys experienced by patients in the former USSR republics of Central Asia. Solzhenitsyn conjures up a memorable image of radiation and radiation therapy in one symbolic sentence, 137 words long (quote in Table 1).

Finally, *Black Rain* by Masuji Ibuse (1969) tells a detailed story of the horrendous effects of radiation inflicted on a large population by nuclear warfare [36]. According to Ibuse, one of the consequences of the atom bomb dropped on Hiroshima was ‘the thundery black clouds that bore down on us … and the rain from them had fallen in streaks the thickness of a fountain pen’ (p. 34). *Black Rain* vividly focuses on radiation in all its medical, social and psychological aspects.

#### 3.1.2 Poems

In *The Radiation Sonnets* (2003), Jane Yolen writes about the radiation therapy that her husband undergoes [37]. In daily entries, she records his (and her) experiences. Her poem *Graduation Day* describes the last day of his radiation therapy (Table 2, for quotes from the cited poems).

**TABLE 2:** Quotations from the cited poems.

The Radiation Sonnets by Jane Yolen [37] On this final day, this graduation, / From the harrowing halls of treatment, / From the harsh teachings of radiation, / Burning as learning (or so the heat meant) / You hold out toward me your secret mask, / That sculpted instrument of torture. / I accept without being asked, / This relic of the cancer culture. / How could I know what went on inside/That hidden, that forbidden room, / Where your skull lay opened wide/To the rays' ever more deadly perfume? / The mask keeps hidden what we most fear. / All I know is that you are still here. (p. 85). Oncological Cocktail by Bonnie St. Andrews [38] Looking too young to bar‐keep the technician comes in shaking a beaker that froths and hisses like volcanic vodka or martinis mixed by Mr. Hyde. Artless as an egg she offers me this cocktail neat or on the rocks and won't take no for an answer. We do not speak of cancer. Ninety proof and guaranteed to knock me sober, this barium refresher is served always with a twist. It smells and tastes like a nuclear waste site laced with a splash of lye. Trying to be philosophical as a Greek and brave as a Roman gladiator I quaff this pewter sludge without flinching, without betraying my mortal fear the fluoroscopy will show my troops have broken rank and an army of mutant cells advances along my exposed flank. … Radiance by Ronnie Sirmans [39] There are wondrous ways to reveal/what's inside our bodies. I learned/this as sickness bloomed and I feared/the scythe shimmering for my mother. / I wore gloves when giving radiation/pills to her at home. At the hospital, / she lay so still for machines' views, / including her PET scan after surgery. / We were told she had to avoid women/who were pregnant and small children/for 3 h after that scan. I stayed/close—we always put each other at risk. / Why did Gabriel herald Mary's radiance, / as if she didn't know what she could bear? /

Bonnie St. Andrews writes poems about her own cancer [38]. In her poem *Oncological Cocktail* (2013), she reflects on the diagnostic and therapeutic steps involved in her cancer treatment, in her *Round & Ripe & Wise* (quote in Table 2).

A recent issue of JAMA included the poem *Radiance* by Ronnie Sirmans (2024), illustrating the burgeoning interest of major biomedical journals in health humanities ([39]; see also [13]).

### 3.2 Music

Radiation and radiation therapy have occasionally been represented in popular music by both mainstream and more innovative artists. Some examples are presented below.

In *Make It Go Away (Radiation Song)* (2018), the American country and folk musician Sheryl Crow sings about her own breast cancer [40]. Its title is repeated 15 times as the last line of the song. Table 3 gives the lyrics to the selected music.

**TABLE 3:** Lyrics from the selected music.

Make it go away by Sheryl Crow [40] I stare into/Some great abyss/And calculate/The things I'd miss/If I could only/Make some sense of this And Madam Butterfly/Resounds/Over the mother‐ship/Her lights flashing around I float above her and/I wonder how/To make it go away/Make it go away/To make it go away/Make it go away I crawl into my circumstance/Lay on the table/Begging for another chance/I was a good girl/I can't understand, how to/Make it go away/Make it go away/Make it go away/Make it go away Sometimes I wonder/Which hurts worse/The thought of dying/Or reliving every hurt/Was love/the illness/And disease the cure/Oh, the cure Make it go away (× 15). Radioaktivität by Kraftwerk [41] Radioactivity Is in the air for you and me Discovered by Madame Curie Tune in to the melody For you and me in space is created Radiates waves to the receiving device When it comes to our future Tune in to the melody Sword of Damocles by Lou Reed [42] I see The Sword of Damocles is right above your head They're trying a new treatment to get you out of bed But radiation kills both bad and good, It cannot differentiate So to cure you, they must kill you The Sword of Damocles hangs above your head

**FIGURE 1:**
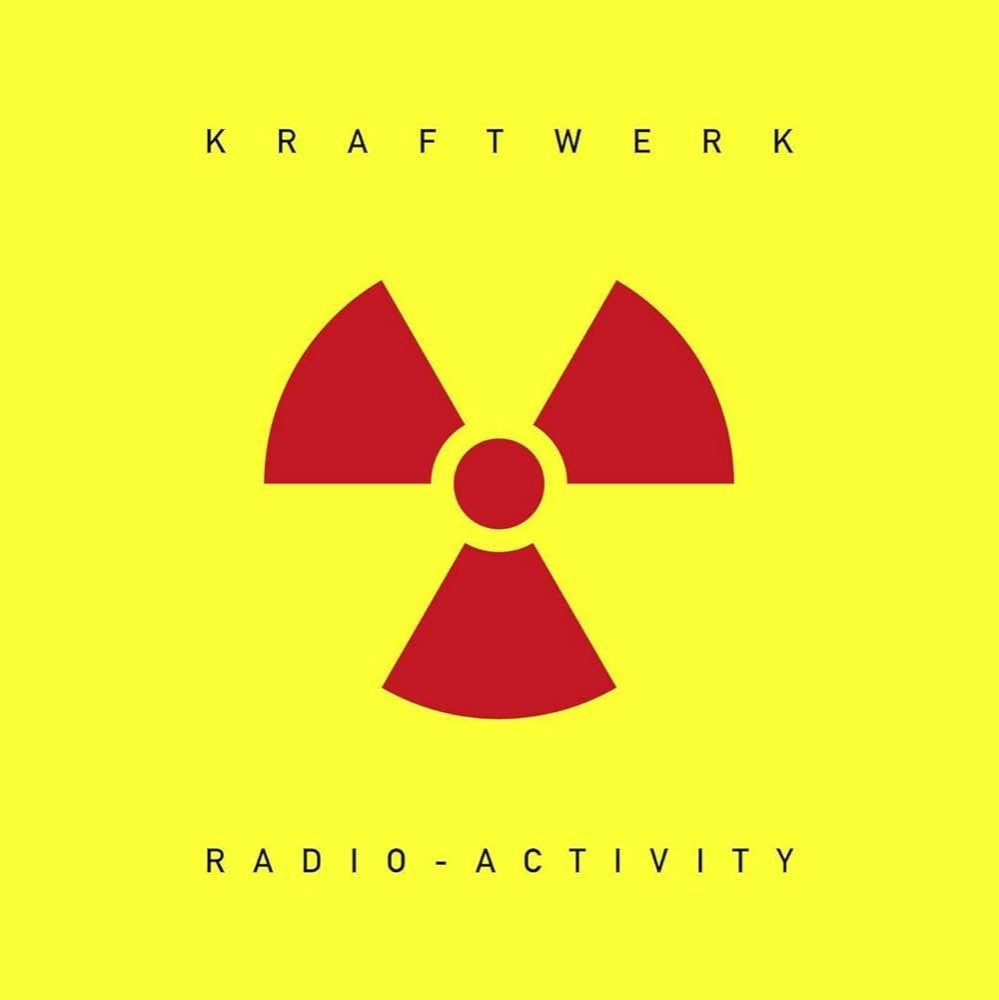
Kraftwerk Radioactivity cover.

In 1975, the German avant‐garde pop group *Kraftwerk* produced an entire album entitled *Radio‐Activity* (see Figure 1), featuring songs such as *Radioactivity*, *Geiger Counter* and *Uranium* [41] (quote in Table 3).

The American rock musician Lou Reed, while himself dying of cancer, wrote the song *Sword of Damocles* (1992) to describe how radiation was part of his medical treatment [42]. Quotes in Table 3.

### 3.3 Films

Wijdick's book *Cinema MD: A History of Medicine On Screen* [26] provides only two movies where radiation therapy is clearly recognisable: *Shadowlands* (1993) [43] and *The Doctor* (1991) [44]. De Fiore et al. list some 100 movies with cancer as a major element—but no movie is identified in that list as focusing on radiation or radiation therapy specifically [25]. The contrast between the representation of radiation and radiation therapy in paintings compared to in movies is somewhat surprising, given the great interest among lay audiences in cancer and its medical management.

Indeed, even in both *Shadowlands* and *The Doctor*, radiation treatment is visible for only a few seconds and in rather stereotypic fashion: a patient in a dark room with apparatus attached around their exposed body.

### 3.4 Paintings

**FIGURE 2:**
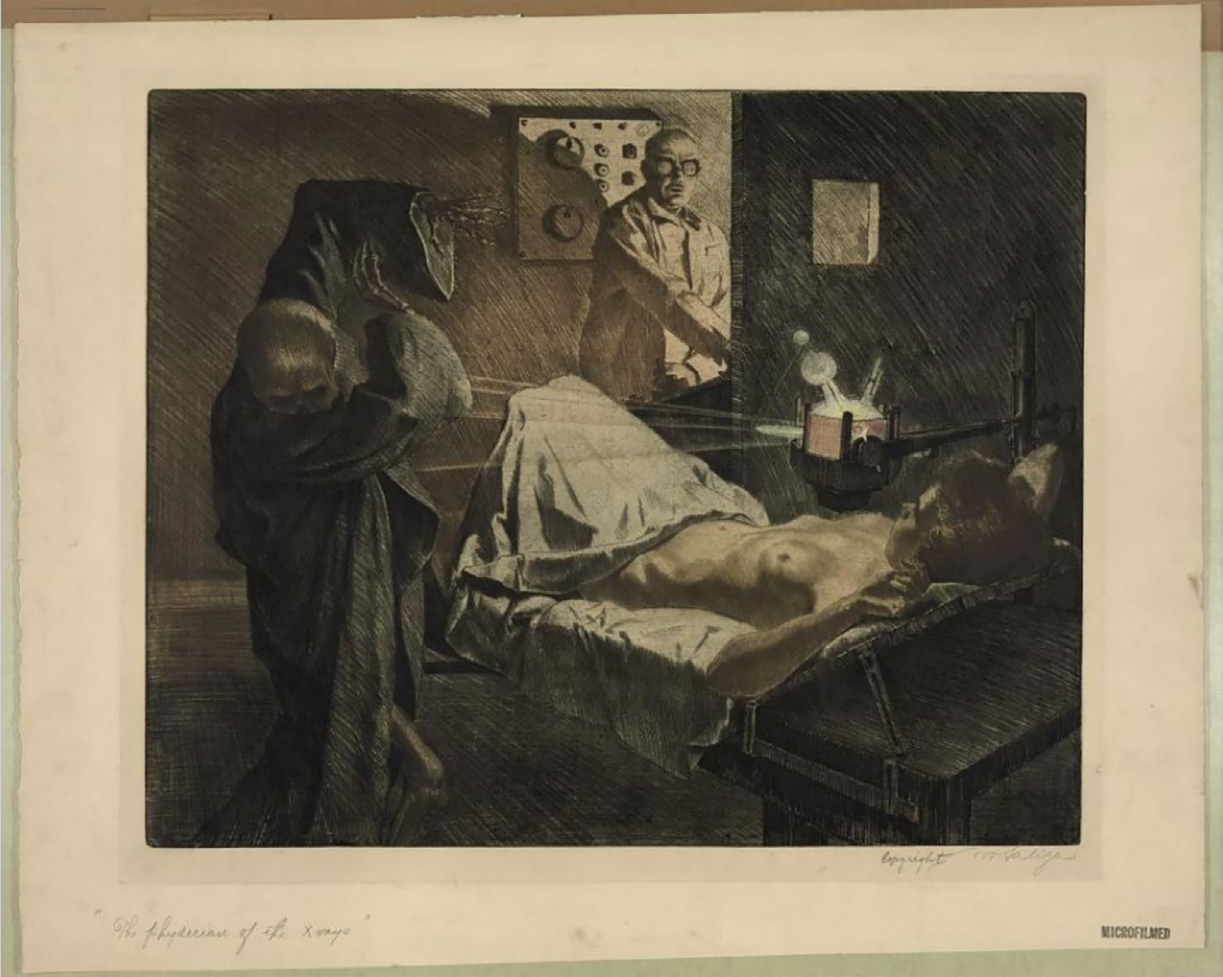
Saliger I. The physician of the X‐rays. Library of Congress Prints and Photographs, Division Washington D.C.; pga 03249//hdl.loc.gov/loc.pnp/pgn.03249, 1920–1940, in the public domain [49].

**FIGURE 3:**
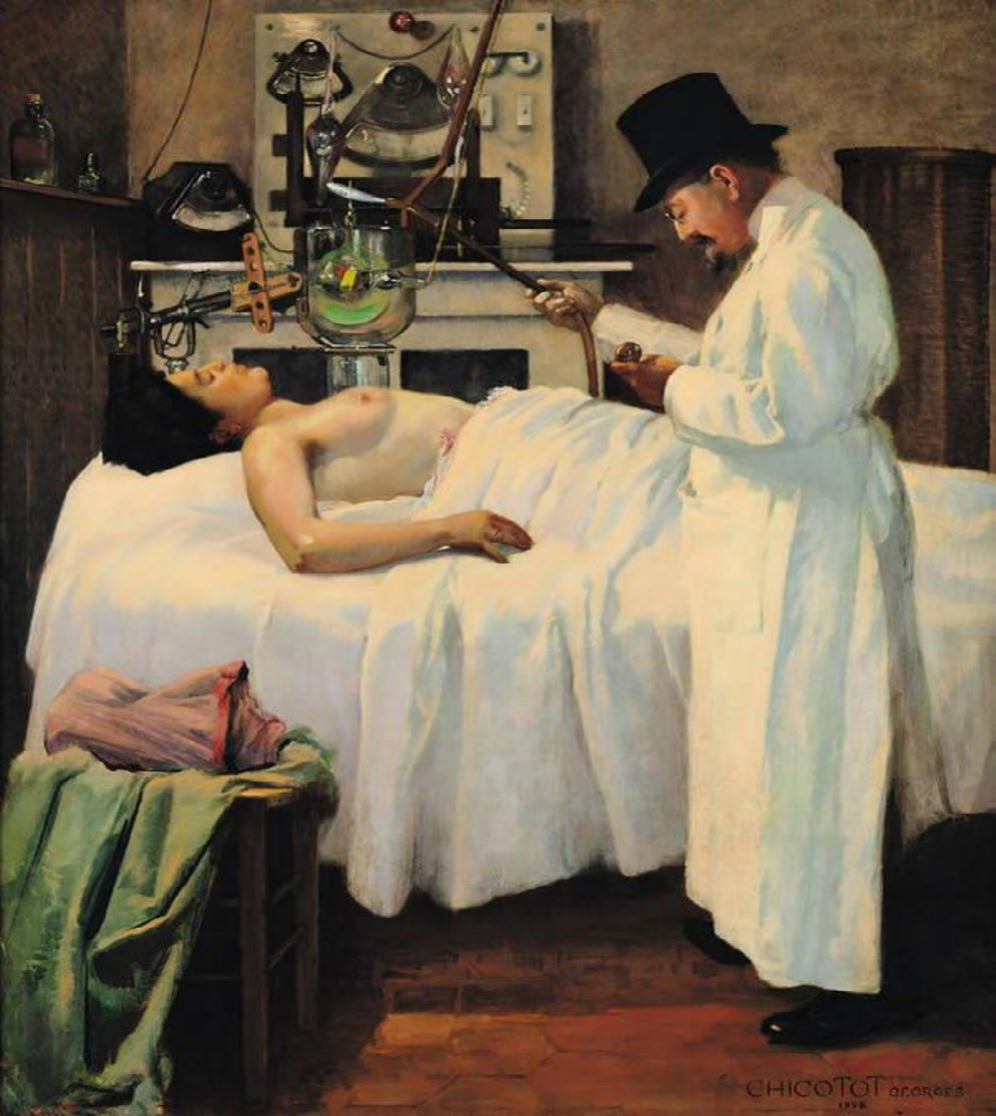
Chicotot G. The first attempt to treat cancer with X‐rays. Paris: Musée de l'Assistance Publique, Hôpitaux de Paris; 1907 ([51], in the public domain).

Paintings by Marcel Duchamp, Pablo Picasso and Georges Braque are discussed by Henderson in her paper on how painters in Europe were challenged ‘to give form to the invisible’ with the advent of X‐rays and radioactivity ([45], cf. [46]). Georges Chicotot, who became renowned as a painter of medical scenes, portrayed the advent of radiotherapy by having his doctor wear a hat, ‘symbolizing professional distance’ [27, 47]. The representation of radiation treatment in the paintings by Robert Pope and Ivo Saliger conveys eerie associations reminiscent of the haunting novels and poems quoted above ([48, 49]; Figure 2). In his graphic novel *Mom's Cancer*, Brian Fies draws his perception of radiation treatment [50]. The first attempt to treat cancer with X‐rays (Figure 3), painted by Chicotot, depicts early 20th century radiation therapy [51].

### 3.5 Academic Research on Patient Perceptions of Radiation Therapy

A selection of empirical studies examining how patients perceive radiation therapy for imaging or treatment is presented below (Table 4; [14, 52, 53, 54, 55, 56, 57, 58, 59, 60, 61, 62]). Rather than conducting a full‐blown systematic review or scoping review, we identified material using a narrative review approach. Given our familiarity with the themes of illness perceptions, quality of life and psychosocial oncology, a sample of recent papers (2016–2025) was selected from high‐impact journals ([27]; Arksey & O'Malley, 2005).

**TABLE 4:** Patients' perceptions of radiation as a therapeutic method.

First author; year; country	Number of patients diagnostic category	Outcome(s) examined	Results
Alashban; 2024; Saudia Arabia [52]	*n* = 412 CT vs. scans	Knowledge and understanding of ionising radiation and dosage; perceived risk of CT‐scan	‘limited understanding of hazards associated with ionising radiation’
ALMasri; 2024; Palestine [53]	*n* = 148 Breast cancer	Cancer‐related fatigue after radiation therapy	FACIT‐F data: intermediate levels of fatigue
Chaballout; 2023; USA [54]	*n* = 46 Anal cancer Chemoradiation (CRT)	Knowledge of CRT; Treatment effects; Stigma; Symptoms	EORTC‐QLQ‐ANL27; RT‐experience ‘hard’; Severe side‐effects
Forbes & Clover; 2023; Australia [55]	*n* = 5 Mask anxiety regarding radiotherapy (RT)	Qualitative questionnaire on radiotherapy perception	Themes: Anxiety triggers, Adjusting, Coping
Gharzai; 2023; USA [56]	*n* = 14 Radiotherapy related adverse effects	Views of patients and health care providers	8‐item adverse effects focused patient reported outcome (PRO) measure
Goldsworthy; 2016; UK [57]	*n* = 3 Focus group on mask wearing	Qualitative themes on radiotherapy perception	Physical comfort; mental perception; passivity
Keast; 2020; Australia [58]	*n* = 20 Head–neck cancer; immobilisation mask	Semi‐structured interview, Thematic analysis on perception of having to wear an immobilisation mask	Themes: Information; Predictors of anxiety; Supportive behaviour and communication with health care providers; Coping
Nixon; 2019; Australia [59]	Mask anxiety in patients with head–neck cancer, *n* = 35	Distress thermometer, and survey to assess perception of mask wearing	Mask anxiety decreased over treatment course; ‘only’ (sic!) 22% experienced anxiety after treatment
Park; 2020; Korea [60]	Fatigue in patients with breast cancer, *n* = 201	FACIT questionnaire and psychological concomitants	Moderate levels of distress
Probst; 2021; UK [61]	Qualitative study in *n* = 9 patients with breast cancer	Studying the experience of radiotherapy	Themes: Information provision; ‘experience of being naked’; disempowerment; health care providers' knowledge
Stewart; 2024; UK [14]	‘ghost in the machine’, *n* = 10 patients with gynaecological cancer	Metaphors of radiotherapy	‘ghosts’ haunting; psychosocial effects; lives not lived
Ulman; 2024; UK [62]	*n* = 14, breast cancer	Qualitative design; experiences of lymphoedema	Symptoms distressing; Often disappointed by health care providers

The papers listed are mostly by scholars working in Anglo‐Saxon/American contexts, and most studies are based on a fairly small number of patients. Diagnostic categories vary. Semi‐structured and qualitative methods of data collection seem to dominate. Results are summarised in the last column. Knowledge, fatigue, anxiety, ‘distress’ and quality of communication with health care providers appear to be the major types of perceptual constructs identified as outcome variables. Illness perceptions depict radiation and radiation therapy as mysterious, scary, invisible, dangerous, anxiety‐provoking, uncontrollable, as also represented in novels, poems, films, music and painting. In the empirical papers, these emotions and cognitions are also dominant findings from the research literature, as shown in this review.

Given the results in these studies, it seems fair to say that radiation therapy is perceived as a quite major stressor, with patients expecting—and not always receiving—support from health care providers [see also [13]].

### 3.6 Academic Research on Interventions

Four systematic reviews have assessed the effects of psychological intervention in patients who are undergoing radiation therapy treatment [63, 64, 65, 66]. Two single studies on this subject will be discussed here as well, as they represent highly interesting examples, one focusing on treatment [67] and the other on outcomes [58]. Results overall are encouraging. Elsner et al. (2017) outline methodological issues with the reviewed studies and state that psychosocial support improves communication and information sharing and can reduce anxiety [63]. Forbes et al. (2023) are highly critical of the studies performed up to now and seem to apply (biomedical) clinical epidemiological criteria in judging the quality of the work available [64]. The use of virtual reality to prepare patients for radiation therapy is reviewed by Grilo et al. (2023), who conclude that such approaches can improve knowledge and reduce distress ([65], see also [59]). O'Connor et al. (2019) review psychological interventions in children [66]. They also are critical of the quality of the studies that they include in their systematic review and conclude that ‘cognitive behavioural approaches appear to be worth exploring further’ (p. 275). Malik et al. (2021) evaluate the effects of pretreatment psychoeducational methods in a large sample of patients (*n* = 1992) with head and neck cancer [67]. Attendance to the ‘prehab’ was associated with a lower frequency of a ‘rocky treatment course’, whereas non‐attendees had a lower overall survival rate (cf. [68]). Victorson et al. (2024) studied the effects of a truly psychological intervention (mindfulness) in patients with prostate cancer, concluding that ‘Audio‐based mindfulness delivered during radiotherapy holds potential to help decrease radiotherapy‐related physical and emotional side effects’ ([69], p. 1).

Music therapy in patients undergoing radiation therapy enjoys a quite substantial research and clinical tradition. A systematic review [55], and a number of individual studies are available. Five individual studies are included in the Forbes et al. (2023) [55] systematic review: [70, 71, 72, 73, 74]. The reviewers conclude that the Chen et al. (2013) [70] and Karadag et al. (2019) [71] studies had an acceptable quality and showed significant improvements in reductions in anxiety, with reductions in anxiety ranging from 10% to 20% [70] to 20% for depression [71, 75].

Three important papers on music therapy were published after the Forbes et al. (2023) systematic review [55]. Lim et al. (2024), using a single‐group crossover design, report music therapy‐related reductions in levels of pain and anxiety in patients undergoing brachytherapy (although no changes in vital signs such as blood pressure, pulse, or respiration rate were observed) [76]. A randomised controlled trial by Toprak et al. (2024) reports similar results: reductions in anxiety, depression and pain [77]. Similarly, a randomised controlled trial in patients undergoing brachytherapy also reports significant reductions in levels of distress and number of symptoms concurrent with music therapy [78]. Alcântara‐Silva et al. (2018) report a randomised controlled trial that was not included in the Forbes et al. (2023) [55] systematic review, in which they found significant reductions in fatigue and depression [79]. Similarly, Rossetti et al. (2017) studied the effects of music on preparation for external beam radiation therapy during simulation in patients with newly diagnosed head and neck or breast cancer; lower levels of anxiety and depression were observed [80]. As summarised by Nardone et al. (2020) ([81], p. 6), ‘Music therapy appears to be a promising non‐pharmacological approach to cope with pain perception and mood disorders in radiotherapy patients’ (see also [82]). Given the available evidence, music therapy appears to be a useful addition in the clinical interventions arsenal for patients who must undergo radiation therapy.

Movie‐making by patients is a fairly recent addition to the therapeutic toolbox [83]. Underlying this approach, where affected patients are actively involved in the management of their illness, is the concept of ‘photovoice’: A participatory method of photography that aims to empower patients to tell their stories using digital equipment, encouraging their self‐efficacy and thereby quality of life. In an Australian study involving children with cancer who had to receive radiation therapy treatment [84], the children's parents produced movies of their radiation therapy experiences. The movies were instrumental in helping families in ‘overcoming fear of the unknown, and increased understanding of treatment; cognitive/attentional distraction helped children maintain self‐control and a positive outlook’ (p. 1). Watching a movie (rather than making one) can also be helpful, especially in paediatric patients. Willis and Barry (2020) describe a case study in which children aged 2 to 6 years watched a DVD movie during radiation therapy, which appeared to reduce the amount of anaesthetic required [85].

Similarly, viewing works of visual art, such as famous paintings, appears to be helpful in reducing anxiety and cancer‐related depression in radiation therapy patients [86]. Viewing paintings of human bodies can also be used as a method to teach and improve the observational skills of medical students. In one study, where first‐year radiology trainees were taught by an artist how to analyse paintings, the trainees exhibited an improvement in their perceptual skills and in their abilities to localise imaging abnormalities in radiographs [87, 88].

## 4 Discussion

Radiation therapy is widely perceived as having both lifesaving and life‐threatening potential. As such, an exploration of how radiation therapy is represented in various art genres, and thus how it is likely to be appreciated by patients and the general population immersed in cultural norms, has scientific, intellectual and clinical value.

In this paper, literature, music, film and paintings were used to highlight how radiation therapy for imaging or treatment is represented in a health humanities perspective. In cancer care, chemotherapy is a relatively frequent subject of medical study and artistic treatment. Radiation therapy, on the other hand, is still a relatively minor topic of interest. The current paper nevertheless identified a niche of recurring radiation and radiation therapy representations within a variety of artistic genres.

A number of novels incorporate radiation and radiation therapy as important and highly meaningful themes. Novelists, poets, musicians, filmmakers and painters have each been inspired by the mysteries and anxieties of radiation and radiation therapy, as reflected in their work. The artistic representation of radiation and radiation therapy has begun to penetrate the scientific community of radiation therapy as well. The empirical studies reviewed in our paper have been published in major radiation therapy and medical journals, as well as in journals of psychology, health humanities and the arts. The references section of our paper is a clear illustration of this point.

### 4.1 Future Research

Overall, our findings can be considered with reference to the stress‐coping model, one of the most applied theoretical approaches in the area of health psychology and health humanities [89]. Summarised in very compact terms, the model proposes a sequence within which the psychological stress of illness is processed by patients, namely: Patients adopt emotional and cognitive representations of their stressor (i.e., their illness); these representations (or ‘illness perceptions’) in turn elicit coping responses; and these coping responses in turn influence the psychosocial consequences of illness, such as quality of life.

Illness perceptions → Stressor → Coping → Quality of Life [53, 54, 55, 56, 58, 60, 62].

Perceived susceptibility [52, 59] emotion focused [14].

Perceived control [61] meaning making [57].

The **novels** by d' Adamo [30] and Gordimer [29] can be viewed as depicting perceived susceptibility, Diski's memoir [31] as depicting perceived control, and the novels by Mann [28] and by Solzhenitsyn [32] as depicting meaning‐making as a coping response.

Similarly, the 12 **studies** from Table 1 may be discussed in terms of the stress‐coping model elements: Alashban et al. [52] and Nixon et al. [59] focus on perceived susceptibility; Probst et al. [61] focus on perceived control; Keast et al. [58] and Stewart et al. [14] focus on emotion‐focused coping; Goldsworthy et al. [57] focus on meaning‐making; and ALMasri et al. [53], Chaballout et al. [54], Forbes et al. [55], Gharzai et al. [56], Park et al. [60], and Ulman et al. [62] each focus on quality of life.

The impact of health humanities on scientific and clinical radiation and radiation therapy continues to grow. Today, the European Society for Medical Oncology recognises the value of supportive and palliative care for patients with cancer [90], as does the American Society of Clinical Oncology [91]. Scientific societies in the area of radiation and radiation therapy embrace similar objectives (ASTRO, ESTRO). As put forward in this paper, empirical evidence supports the use of therapeutic approaches from the health humanities domain for the benefit of patients who must undergo radiation therapy. Anxiety and depressed emotion can be addressed by behavioural methods resulting in improvements in self‐efficacy, levels of fatigue, quality of sleep and quality of life. Malik et al. even suggest that positive outcomes may in turn contribute to enhanced survival [67].

Given the empirical work on perceptions of radiation for imaging (radiology) and for cancer treatment (radiation therapy), a number of **research** suggestions can be formulated.

1. Integrating theoretical models and empirical expertise from the health humanities will help in the development and delivery of instrumentally useful interventions for patients undergoing radiation therapy [92].
2. Developing condition‐specific and therapeutic approach‐specific questionnaires to assess the adverse effects associated with radiation therapy will be helpful in improving the quality of care for patients undergoing radiation therapy [13, 56].
3. Health care providers must be supported to incorporate health humanities theoretical models and empirical interventions in improving quality of life and quality of care in radiation treatment (e.g., [93]).
4. Incorporating patients' stories about radiation by encouraging expressive writing, drawings and photovoice studies will improve the quality of care for radiation therapy patients [94, 95].
5. Integrating behavioural expertise in patient‐linked research and care is a sine qua non for the further development of quality medical care for persons having to undergo radiation treatment.

### 4.2 Limitations

Our paper has an important limitation in being constrained by cultural balkanisation. While we were able to read and assess papers and works published in a number of languages (including English, French (e.g., [96]), German (e.g., [22, 28]), Swedish (e.g., [97]) and Dutch), works published in other major languages (such as Spanish or Chinese) were beyond the scope of the current authors. Similarly, the genres of music or visual arts within our purview were largely those of the so‐called Western tradition, or which have found favour within Western audiences. Future study of how radiation and radiation therapy are depicted in (say) Asian or African cultures will be essential for broadening our understanding and for fully exploiting the insights to be gained from human artistic expression.

### 4.3 Clinical Implications

Papers on doctor–patient communication have long shown how patient‐centred communication is an important predictor of outcomes in radiation therapy [13, 98]. In their review, Flores et al. identify this prehabilitation as ‘a proactive approach to patient care focused on optimising physical, mental, and psychological well‐being before patients undergo a medical treatment’ ([99], p. 83). They conclude that appropriate ‘prehab’ has the potential to reduce morbidity and rightly point at the relative underrepresentation of interventions informed by medical/health humanities and the relative overrepresentation of more‐or‐less biomedical approaches (e.g., physical exercise, technology apps, etc.).

Clinical implications follow from the literature reviewed, and from the clinical applications of health humanities work in other medical fields (e.g., oncology). In the context of health humanities, a brief reference to the seminal paper by Peabody ([100]) is warranted:

‘The good physician knows his patients through and through, and his knowledge is bought dearly. Time, sympathy, and understanding must be lavishly dispensed …. One of the essential qualities of the clinician is interest in humanity, for the secret of the care of the patient is caring for the patient’ (p. 882). Radiation oncologists and radiation therapists are often separated from contact with patients by thick concrete and lead walls—the very act of their medical intervention takes place in complete separation from the patient. Nevertheless, empirical studies provide a quite impressive list of therapeutic implications in the radiotherapy setting.

Linked to these research suggestions, a number of **clinical implications** may be formulated.

○ Use Patient Reported Outcomes (PROs) systematically: continuous assessment of major psychological problems (anxiety, panic, dissatisfaction; [6, 101, 102, 103]),
○ Involve patients in developing supportive measures to reduce psychological morbidity (anxiety, panic),
○ Explore illness perceptions in the clinical encounter (e.g., [13]),
○ Address unhelpful illness perceptions and treatment beliefs (e.g., [7]),
○ Teach patient skills in a cognitive behavioural therapy format (i.e., relaxation skills, mindfulness, coping training, breathing exercises, stress management, [13]),
○ Use modern technology (e.g., virtual reality, [65, 104, 105]),
○ Apply music therapy [70, 106],
○ Improve health care provider—patient communication skills via structured training [106],
○ Improve knowledge, via the provision of information [55, 65],
○ Involve radiation therapy societies and establish a health humanities niche in the major scientific journals [10, 106, 107, 108]; encourage opinion leaders and expert patients to present blogs on biopsychosocial care, with the overall objective of addressing perceptions of radiation therapy, and improving the image of radiotherapy: from ‘scary, mysterious’ to ‘healing’.

## 5 Conclusion

This paper aimed to shed light on the depiction of radiation and radiation therapy in various art genres as identified in empirical studies, in a health humanities context: overall, novels, poems, music, film and painting depict radiation and radiation therapy as associated with fear, mystery and fascination. Our findings help contribute to a deeper understanding of health humanities offering diagnostic and therapeutic approaches that address and reduce fear, improving quality of life and quality of medical care via medical and psychological methods. A rich array of research and clinical implications arise.

## Conflicts of Interest

The authors declare no conflicts of interest.

## Data Availability

The data that support the findings of this study are available from the corresponding author upon reasonable request.
